# The Impact of Mental Fatigue on Decision-Making Abilities, Visual Search Strategies, and Simple Reaction Time in Handball Players: A Randomized Crossover Study

**DOI:** 10.3390/sports14040128

**Published:** 2026-03-25

**Authors:** Jeongwon Kim, Dongwon Yook, Sojin Han

**Affiliations:** Department of Physical Education, Graduate School, Yonsei University, Seoul 03772, Republic of Korea; jw31244@yonsei.ac.kr (J.K.); dyook@yonsei.ac.kr (D.Y.)

**Keywords:** decision-making, handball, mental fatigue, visual search behavior

## Abstract

This study investigated the effects of mental fatigue induced by social media (SM) use and the Stroop task on decision-making, visual search strategies, and reaction time in elite collegiate handball players (*n* = 16). Using a randomized, counterbalanced cross-over design, both interventions successfully induced subjective mental fatigue, as confirmed by visual analog scale (VAS) ratings. Decision-making accuracy and reaction time improved following the Stroop task, likely due to compensatory mechanisms described in the regulatory-control model. In the SM condition, no significant impairments were observed in decision-making performance; however, visual reaction time was specifically delayed, while auditory reaction time remained unaffected, suggesting modality-specific effects of SM-induced fatigue. Visual search behaviors remained largely stable, with only marginal alterations observed in non-task-relevant areas following the Stroop task. These findings highlight the cognitive resilience and adaptive control mechanisms of elite athletes in maintaining and, in some cases, enhancing performance under mental fatigue. Future studies should integrate neurophysiological indices and manipulate motivational factors to further clarify these mechanisms across diverse athletic populations.

## 1. Introduction

Mental fatigue (MF) is a psychophysiological state characterized by feelings of tiredness and reduced energy typically occurring after prolonged cognitive activity [1,2]. Previous studies have demonstrated that mental fatigue negatively affects both cognitive and physical performance [3,4,5], including impairments in attention, reaction time, accuracy, and visual perception [2]. Several investigations report that mental fatigue deteriorates decision-making in team-sport athletes [4,6,7]. Decision-making is a critical skill for successful performance in dynamic and unpredictable sport environments [6,8], as it relies heavily on the brain’s capacity to effectively extract contextual information from complex visual scenes [9,10]. Traditionally, studies have induced mental fatigue using cognitively demanding tasks such as the Stroop task, and subsequently evaluated its effects on sport performance during training or simulated games [4,6]. However, ecological validity in sport remains a concern [11]. Recent studies have therefore used more ecologically relevant tasks, including social media and video games [12,13]. It has been reported that more than 30 min of smartphone use can induce mental fatigue in soccer players [8], and that both Stroop and social media tasks increase fatigue without clear differences in muscular endurance [14]. These findings suggest that the characteristics and ecological relevance of fatigue-inducing tasks may lead to different behavioral outcomes. Therefore, understanding how different fatigue-induction methods influence athletes’ decision-making is crucial. Decision-making is supported by specific visual search strategies and perceptual–cognitive skills [15,16], but the effects of mental fatigue on visual search appear to be sport-specific. For example, in basketball, changes in fixation frequency but not duration have been reported [13], whereas in soccer simulations, fewer fixation sequences have been observed, with modest effect sizes [4]. Most existing research has focused on soccer [4,6], swimming [17,18], and cycling [19,20], leaving a gap in handball. Handball requires tracking multiple moving players and making rapid, context-dependent decisions [21]. A previous study suggested that affective states may impair decision-making in handball players [22], yet little is known about how mental fatigue influences decision-making and visual search in this sport.

Reaction time is another key component of performance in team sports, reflecting the ability to respond quickly and appropriately, to auditory or visual stimuli [23]. Because athletes process multisensory information [24], both modalities are relevant for understanding responsiveness. Although auditory reaction time is typically faster than visual reaction time [25], results remain inconsistent [26,27]. In handball, players must react promptly to both auditory cues (e.g., instructions from teammates or referees) and visual cues (e.g., opponents’ movements), making cross-modal comparisons important. The present study aimed to examine the effects of mental fatigue induced by social media use and the Stroop task on decision-making performance in handball players. It was hypothesized that mental fatigue would impair decision-making speed and accuracy, and alter visual search strategies in handball players.

## 2. Materials and Methods

### 2.1. Participants

Sixteen male collegiate handball players (age: 20.8 ± 1.6 years; height: 181.8 ± 6.6 cm; body mass: 80.4 ± 8.6 kg; visual acuity: 1.1 ± 0.6) participated in this randomized, counterbalanced crossover study. All competed in the Korean University Handball League with over 10 years’ experience (10.4 ± 1.6 years). Sample size was calculated using G*Power 3.1.9.2 (University of Kiel, Kiel, Germany) for a repeated-measures ANOVA (two conditions × two measurements) based on an expected large effect size (ηp^2^ = 0.20), power = 0.90, α = 0.05, yielding a required minimum of 14 participants. Considering potential dropouts, 16 participants were recruited.

All procedures conformed to the Declaration of Helsinki and were approved by the Institutional Review Board (IRB No. 7001988-202406-HR-2298-02). Written informed consent was obtained from all participants.

### 2.2. Experimental Overview

**Figure 1:**
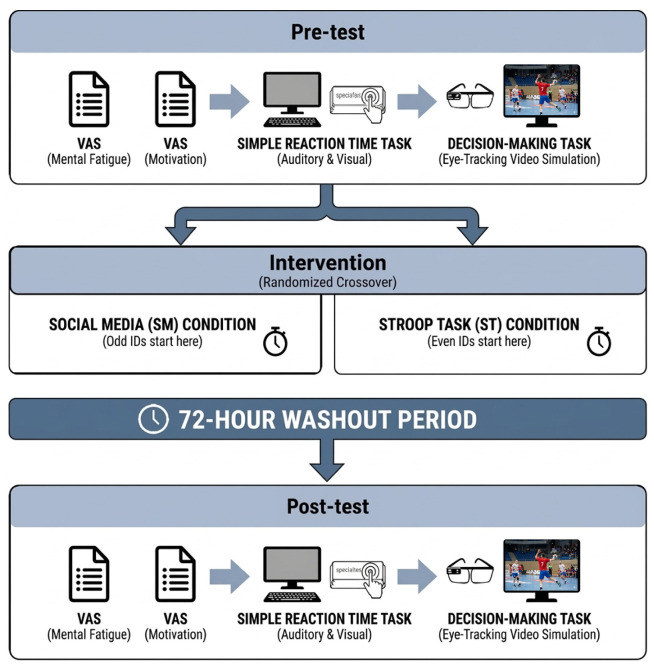
Experimental design.

The procedure is illustrated in Figure 1. Mental fatigue was induced using two conditions—social media (SM) and Stroop task (ST)—in a randomized crossover design in which all participants completed both [28,29]. Sessions were separated by a 72 h washout period. Participants with odd ID numbers began with SM and even-numbered participants with the ST condition. Before testing, participants were instructed to sleep ≥ 8 h, avoid alcohol and strenuous exercise for 24 h, refrain from caffeine on the testing day, and eat a moderate meal 1.5 h before. Upon arrival, they completed visual analog scales (VASs) for mental fatigue and motivation, followed by computer-based simple reaction time tasks (auditory and visual) and an eye-tracked handball decision-making task. After calibration and three familiarization trials, participants viewed 10 randomized clips, each pausing before a pass to identify the optimal direction as quickly and accurately as possible. Participants then completed the assigned intervention under supervision and repeated the same sequence.

### 2.3. Mental Fatigue Protocols

In the SM condition, participants browsed Instagram for 30 min on a smartphone according to a previously described protocol [8]. To minimize variability in platform use, access was restricted to Instagram (Meta Platforms, Inc., Menlo Park, CA, USA; https://www.instagram.com), and participants were monitored to prevent access to other applications. In the ST condition, participants completed a computerized Stroop task using E-Prime 3.0 (Psychology Software Tools, Inc., Sharpsburg, PA, USA). Consistent with a previous study [29], the words green, red, yellow, and blue appeared in incongruent font colors on a black background. Participants pressed the corresponding key (G, R, Y, B) as quickly and accurately as possible, ignoring word meaning. Each word appeared individually for 1000 ms in 18-point font.

### 2.4. Outcome Measure

#### 2.4.1. Manipulation Check

Subjective mental fatigue and motivation were assessed with 100 mm visual analog scales (VASs) [28,29]. The mental fatigue scale ranged from “not fatigued at all” to “extremely fatigued,” and the motivation scale from “very low” to “very high.” Both scales were administered before and after each intervention.

#### 2.4.2. Decision-Making Task

The task assessed decision-making accuracy and reaction time in offensive handball scenarios based on a previous study [4]. It was implemented as a computer-based video simulation. Participants viewed 10 offensive clips on a 0.95 m × 0.54 m monitor (model: 40MB27HM; LG Electronics, Seoul, Republic of Korea) positioned 1 m away. Each video paused 1.5 s before the pass action, and participants then had 10 s to select the most effective of three passing options. The next clip began immediately after each selection.

Videos were extracted from official International Handball Federation (IHF) matches and edited with GOM Mix Pro (version 1.0.1.1452; GOM&Company, Seoul, Republic of Korea). Two former professional handball players and one collegiate coach verified the scenario validity. Performance was scored on a 3-point scale (3 = optimal, 2 = acceptable but suboptimal, 1 = inefficient).

Decision-making accuracy and reaction time (ms) were collected using PsychoPy4 (v2023.2.3; Open Science Tools Ltd., Nottingham, UK) and stored as CSV files for analysis.

#### 2.4.3. Simple Cognitive Task

Following the VAS assessment, participants performed a simple reaction time task programmed in E-Prime. The same 0.95 m × 0.54 m monitor was positioned 1 m in front of the participants. After three practice trials, five valid trials were recorded for each modality. In the visual reaction time task, participants pressed the “1” key as quickly as possible when a star symbol (★) appeared on the screen. In the auditory reaction time task, a whistle sound was presented as the auditory stimulus. Participants pressed “1” immediately upon hearing the sound. The reaction time was defined as the interval between stimulus onset and key press, measured in milliseconds (ms), and the mean of the five valid trials per condition and session was used for analysis.

#### 2.4.4. Visual Search Strategies

Gaze behavior was recorded using the Dikablis Eye Tracking System (Ergoneers GmbH, Manching, Germany), a head-mounted system that records eye movements using infrared reflection technology. Reflected light was transmitted to the analysis computer, enabling real-time gaze visualization. Calibration was performed before each trial to ensure spatial accuracy. The sampling rate was 60 Hz, and gaze coordinates were captured in two dimensions (x, y) with a resolution of 648 × 488 pixels. Data were processed in D-Lab software (version 3.52; Ergoneers GmbH, Manching, Germany). Fixations were analyzed for duration and spatial distribution. Video sequences were segmented into 1.5 s intervals prior to the pass action. The fixation duration rate was calculated as the proportion (%) of total fixation time relative to total task duration. Fixation location analysis quantified the proportion (%) of total fixation duration allocated to five areas of interest: ball holder, attacker, defender, goalkeeper, and other regions [30].

### 2.5. Statistical Analysis

Statistical analyses were performed using R (version 4.5.2; R Foundation for Statistical Computing, Vienna, Austria). Linear mixed-effects models (LMMs) were used to examine the effects of Condition (SM vs. ST), Time (Pre vs. Post), and their interaction on the visual analog scale, decision-making abilities, simple reaction time, and visual search behavior. Participant ID was included as a random intercept to account for repeated measurements within individuals. Fixed effects include Condition, Time, and their interaction. To determine the most appropriate covariance structure, three candidate structures—compound symmetry (CS), first-order autoregressive (AR(1)), and unstructured (UN)—were compared. Models were fitted using restricted maximum likelihood (REML) and evaluated using the Akaike Information Criterion (AIC), and the model with the lowest AIC was selected. Effect sizes (ηp^2^) were interpreted as small (<0.03), medium (0.03–0.10), large (0.10–0.20), or very large (≥0.20) [31].

Fixation location distributions were analyzed separately using paired t-tests to examine Pre-to-Post differences within each condition (SM and ST). Normality of the difference scores was assessed using the Shapiro–Wilk test. Statistical significance was set at *p* < 0.05 for all analysis.

## 3. Results

### 3.1. Visual Analog Scale

**Figure 2:**
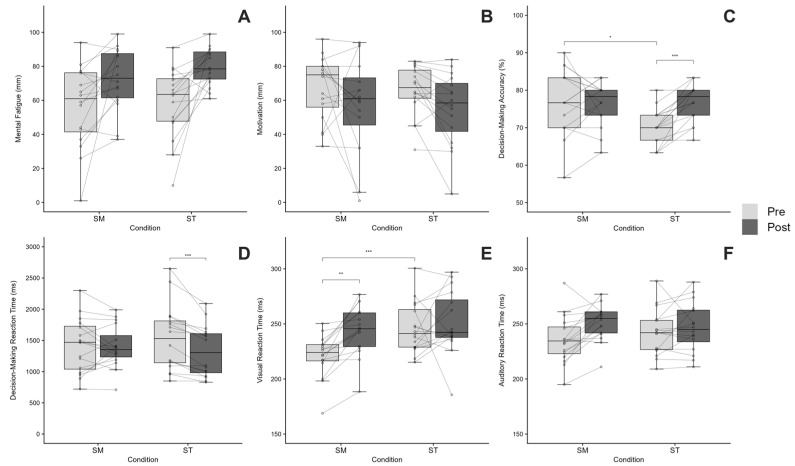
Effects of mental fatigue induced by SM and ST condition on subjective ratings, decision-making performance, and simple reaction time. (**A**) Mental fatigue, (**B**) motivation, (**C**) decision-making accuracy, (**D**) decision-making reaction time, (**E**) visual reaction time, and (**F**) auditory reaction time measured before (Pre) and after (Post) the intervention. Individual data are shown as gray dots connected by lines, while boxplots indicate the group median and interquartile range (IQR). (* *p* < 0.05, ** *p* < 0.01, *** *p* < 0.001; Bonferroni-adjusted pairwise comparisons).

Mental fatigue showed no significant Condition × Time interaction (*F*(1, 45) = 0.30, *p* = 0.585, ηp^2^ = 0.007), nor a significant main effect of Condition (*F*(1, 45) = 0.41, *p* = 0.524, ηp^2^ = 0.009). However, a significant main effect of Time was observed (*F*(1, 45) = 18.82, *p* < 0.001, ηp^2^ = 0.295), indicating higher fatigue levels after the interventions than before (Figure 2A). Motivation showed no significant Condition × Time interaction (*F*(1, 45) = 0.01, *p* = 0.921, ηp^2^ = 0.001), nor a main effect of Condition (*F*(1, 45) = 0.15, *p* = 0.704, ηp^2^ = 0.003). However, a significant main effect of Time was observed (*F*(1, 45) = 6.65, *p* = 0.013, ηp^2^ = 0.129), indicating lower motivation levels after the interventions than before (Figure 2B). Detailed results are presented in Table 1.

### 3.2. Decision-Making Abilities

Decision-making abilities revealed a significant Condition × Time interaction for both variables (accuracy: *F*(1, 45) = 8.24, *p* = 0.006, ηp^2^ = 0.155; reaction time: *F*(1, 45) = 5.07, *p* = 0.029, ηp^2^ = 0.101). For accuracy, significant main effects were found for both Condition (*F*(1, 45) = 7.82, *p* = 0.008, ηp^2^ = 0.148) and Time (*F*(1, 45) = 7.75, *p* = 0.008, ηp^2^ = 0.147). Bonferroni-adjusted pairwise comparisons indicated that accuracy significantly increased from Pre to Post (76.9%) in the ST condition (*p* < 0.001), whereas no significant change was observed in the SM condition (Figure 2C). For reaction time, a significant main effect of Time was observed (*F*(1, 45) = 15.10, *p* < 0.001, ηp^2^ = 0.251), whereas the main effect of Condition was not significant (*F*(1, 45) = 2.64, *p* = 0.112, ηp^2^ = 0.055). Bonferroni-adjusted pairwise comparisons showed that reaction time significantly decreased from Pre- to Post-test in the ST condition (*p* < 0.001), while no significant change was observed in the SM condition (Figure 2D). Detailed results are presented in Table 1.

### 3.3. Simple Reaction Time

A significant Condition × Time interaction was found for visual reaction time (*F*(1,45) = 4.24, *p* = 0.045, ηp^2^ = 0.086), along with significant main effects of Condition (*F*(1, 45) = 15.54, *p* < 0.001, ηp^2^ = 0.257) and Time (*F*(1, 45) = 8.38, *p* = 0.006, ηp^2^ = 0.157). Bonferroni-adjusted pairwise comparisons revealed that visual reaction time significantly increased from Pre to Post in the SM condition (*p* = 0.001), whereas no significant change was observed from Pre to Post in the ST condition. Additionally, SM exhibited a significantly shorter visual reaction time compared to ST at Pre (*p* < 0.001), but this difference disappeared at Post (Figure 2E). For auditory reaction time, the Condition × Time interaction was not significant (*F*(1, 45) = 3.28, *p* = 0.077, ηp^2^ = 0.068), nor was the main effect of Condition (*F*(1, 45) = 0.29, *p* = 0.595, ηp^2^ = 0.006). However, a significant main effect of Time was observed (*F*(1, 45) = 10.13, *p* = 0.003, ηp^2^ = 0.184), indicating longer reaction times at Post compared with Pre (Figure 2F).

### 3.4. Visual Search Behavior

**Figure 3:**
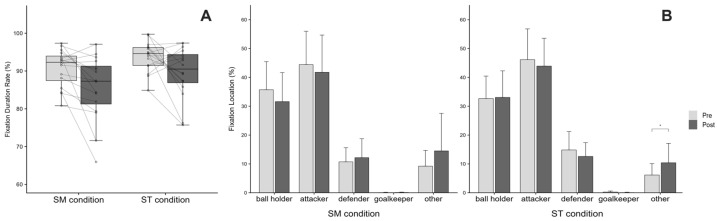
Fixation duration rate and location distributions across conditions. (**A**) Fixation duration rate (%) from Pre to Post for each condition. Individual data are shown as gray dots connected by lines, while boxplots indicate the group median and interquartile range (IQR). (**B**) Fixation location distributions (%) across areas of interest (AOIs): ball holder, attacker, defender, goalkeeper, and other, for the SM (left) and ST (right) conditions. Error bars indicate the standard error (SE). * *p* < 0.05 (paired *t*-test for Pre-to-Post changes within the ST condition).

Fixation duration rate showed no significant Condition × Time interaction (*F*(1, 45) = 0.10, *p* = 0.754, ηp^2^ = 0.002). However, significant main effects were observed for both Condition (*F*(1, 45) = 7.18, *p* = 0.010, ηp^2^ = 0.138) and Time (*F*(1, 45) = 12.96, *p* = 0.001, ηp^2^ = 0.224). Specifically, fixation duration was significantly longer in the ST condition compared to the SM condition. Furthermore, fixation duration decreased from Pre-to-Post (Figure 3A). Regarding fixation location, in the SM condition, paired t tests revealed that fixations were distributed primarily toward the attacker, ball holder, defender, goalkeeper, and other regions, in that order. No significant Pre-to-Post differences were found for any region (all *p* > 0.05; Figure 3B, left panel). In the ST condition, the same fixation order was observed; however, only the other area showed a significant Pre to Post difference, *t*(15) = −2.25, *p* = 0.050 (Figure 3B, right panel).

## 4. Discussion

This study examined the effects of mental fatigue induced by social media use and the Stroop task on decision-making, visual search behavior, and simple reaction time in collegiate handball players. Both interventions increased subjective mental fatigue, while motivation showed an overall decreasing trend overtime.

Contrary to our hypothesis, decision-making accuracy improved following the Stroop task. This finding contrasts with several previous studies reporting that mental fatigue impairs sport-specific decision-making performance in soccer and combat-sport athletes [4,6,13]. However, some studies have suggested that athletes may maintain or even improve performance under cognitive strain through compensatory control mechanisms [32,33]. This finding can be interpreted within Hockey’s motivational control model of cognitive fatigue, which posits that compensatory effort can be mobilized to maintain performance under increased demands [33]. As previously argued in this framework, increased effort allocation can stabilize core task performance despite high workloads. Notably, the present study observed a tendency for performance to be maintained or even improved over time, despite a concurrent increase in mental fatigue and a decline in motivation. This suggests that performance outcomes cannot be fully explained by psychological states alone and it is plausible that participants may have mobilized additional cognitive resources during task execution to partially compensate for the effects of fatigue. However, given that the use of identical video stimuli were used in both the pre- and post-test, the possibility that learning effects resulting from repeated exposure partially contributed to the improvement in decision-making accuracy cannot be entirely excluded. Increased familiarity with the same stimuli may have enabled participants to process and judge situation more rapidly and accurately. Therefore, caution is warranted when interpreting the observed improvement in performance following the Stroop task.

The finding that social media use did not impair decision-making performance differs from previous studies. Previous studies have reported that smartphone use deteriorates decision-making in soccer [8] and basketball players [13]. However, the present study conducted with handball players yielded results inconsistent with these findings. In this regard, several studies have similarly reported an absence of performance decrements in isometric or endurance tasks [17,34,35]. These discrepancies may be attributed to differences in task characteristics and the level of cognitive processing required. From the perspective of the regulatory-control model, it is also plausible that additional cognitive effort was recruited to counteract negative effects and maintain performance even under mental fatigue. Furthermore, the fact that the participants in this study were elite handball players is an important consideration. Through prolonged training and competitive experience, elite athletes may develop optimized decision-making strategies and efficient information-processing schemas. It is plausible that these characteristics contributed to the preservation of decision-making performance under mental fatigue condition. Although this study did not include a control or amateur comparison group, future research incorporating different expertise levels would allow to clarify group differences in decision-making performance under mental fatigue.

Visual reaction time was significantly slower after social media use but not after the Stroop task. This could be attributed to the monotonous nature of the visual task, inducing boredom and reduced engagement [36]. Importantly, the delay in visual reaction time following social media use has meaningful practical implications. Given the demands of handball, in which players must respond instantaneously to opponents’ movements and ball trajectories, even a modest decline in reaction time induced by 30 min of social media use (e.g., Instagram) could result in conceding goals or having critical passes intercepted during actual match play. These findings may serve as a reference for developing coaching strategies related to the management of athletes’ smartphone and social media use prior to competition. In contrast, the Stroop task likely maintained cognitive engagement, leading to compensatory activation that preserved performance. Auditory reaction time remained unaffected, likely reflecting faster processing along auditory pathways compared with visual pathways [25,37]. This aligns with evidence that auditory information accelerates visuomotor reactions in elite athletes under multisensory conditions [37]. As such, auditory tasks may demand lower cognitive processing, making them less susceptible to mental fatigue. These finding suggest that under conditions of mental fatigue, such as those experienced in the latter stages of a match, athletes may respond more reliably to auditory cues than to visual information. This implies that coaching strategies prioritizing auditory stimuli—such as verbal instruction or whistle signals—may prove particularly effective.

In terms of visual search strategies, fixation duration remained unchanged, consistent with basketball studies reporting preserved fixation duration despite mental fatigue [12,28]. Studies showing altered fixation duration often involve sport-specific occlusion tasks [38] or more complex tactical contexts [4]. In basketball, Faro et al. [12] reported changes in fixation frequency but not duration, further suggesting that fixation duration is a relatively stable index of visual search behavior under mental fatigue, particularly in elite athletes with high task familiarity. The handball scenarios were likely highly familiar to elite players, limiting cognitive load. The only significant change occurred in “other” fixation areas after the Stroop condition, suggesting a shift in attentional focus rather than a change in information-processing strategy. According to previous studies [39], mental fatigue can shift attention from goal-directed to stimulus-driven processing, potentially causing inattentional blindness [16,40]. However, since accuracy improved, it is likely that participants compensated effectively for this shift.

In summary, the findings of this study indicate that elite handball players are able to maintain, and in some cases even improve, performance under condition of mental fatigue. Despite increased mental fatigue and a general decline in motivation, performance was preserved, indicating that performance regulation is not attributable to a single factor but rather reflects the operation of multifaceted cognitive control mechanisms. These results provide practical insights for coaches, indicating that subjective fatigue does not necessarily translate into immediate performance decrements. Nevertheless, since the present study was conducted in a laboratory setting using video-based stimuli and simple reaction tasks, it may not fully capture the multisensory processing and complex motor demands of actual match situations. Therefore, future research should aim to validate these findings in more ecologically valid environments or simulation-based settings that closely replicate real-world competitive contests.

## 5. Limitations and Future Directions

Several limitations should be noted. First, identical video stimuli were used in both the pre-and post-test. Repeated exposure to the same stimuli may increase familiarity and consequently influence performance outcomes, compromising internal validity [29]. Future research should minimize this confound by employing different video clips of comparable difficulty. Second, mental fatigue was assessed solely using the Visual Analog Scale (VAS). Although the VAS offers practical utility as a rapid field-based measurement tool, its ability to clearly distinguish between mental and physical fatigue may be limited [41], thereby constraining its reliability as a standalone subjective index. Previous research has demonstrated that subjective fatigue is closely associated with neurophysiological markers, such as changes in frontal alpha activity and heart rate variability [42,43]; however, the VAS does not directly capture these underlying processes. Therefore, future studies should incorporate objective physiological measures to more precisely elucidate the neurophysiological mechanisms underlying mental fatigue. Third, the present study was conducted with a sample of 16 collegiate elite handball players, which limits the generalizability of the findings to other populations, such as amateur athletes or players from other sports. In addition, the present study focused on area-of-interest (AOI)-based visual search analyses to examine visual search characteristics under mental fatigue, without considering positional roles or tactical differences among players. Future research employing larger samples with position-specific analyses would enable the development of practically applicable guidelines tailored to the distinct role requirements of each position in handball.

## 6. Conclusions

Both social media use and the Stroop task effectively induced mental fatigue and resulted in an overall decline in motivation levels in elite handball players. Contrary to expectations, only the Stroop condition improved decision-making performance, which may be explained by the combined influence of compensatory cognitive control mechanism and potential learning effects resulting from repeated exposure to identical stimuli. Social media use did not impair performance, suggesting that not only task characteristics but also the high level of expertise and strategic cognitive regulation inherent to elite athletes may have buffered against performance decrements. Mental fatigue selectively impaired visual, but not auditory, reaction time, likely reflecting differences in sensory processing demands. Furthermore, fixation behavior remained largely unchanged, except for minor alterations in non-task-relevant regions. These findings suggest that elite handball players are capable of maintaining performance under mental fatigue, potentially through adaptive cognitive regulatory mechanisms. From an applied perspective, these findings provide a basis for coaching strategies, such as regulating pre-game smartphone use and emphasizing auditory cues during performance. Future studies should further investigate these mechanisms across different levels of expertise and in more ecologically valid environments.

## Figures and Tables

**Table 1:** Descriptive statistics of all variables by condition and time.

Variable	Condition	Pre (Mean ± SD)	Post (Mean ± SD)	Δ (95% CI)	*p*
Mental Fatigue (VAS)	SM	56.38 ± 24.20	72.50 ± 18.31	16.12 (3.53–28.72)	0.013
	ST	58.44 ± 21.03	79.56 ± 11.03	21.13 (8.53–33.72)	0.002
Motivation (VAS)	SM	67.56 ± 18.71	57.38 ± 28.12	−10.19 (−25.75–5.37)	0.194
	ST	65.81 ± 14.99	55.00 ± 21.34	−10.81 (−19.28–−2.34)	0.014
Decision-making Accuracy (%)	SM	77.08 ± 8.93	76.67 ± 5.96	−0.42 (−3.78–2.94)	0.804
	ST	70.21 ± 4.79	76.88 ± 4.94	6.67 (3.31–10.03)	<0.001
Decision-making Reaction Time (ms)	SM	1417.50 ± 440.01	1400.62 ± 336.34	−16.88 (−149.61–115.86)	0.799
	ST	1534.38 ± 529.29	1330.00 ± 409.42	−204.38 (−296.90–−111.85)	<0.001
Visual Reaction Time (ms)	SM	220.93 ± 19.50	244.24 ± 22.71	23.31 (9.97–36.65)	0.001
	ST	246.39 ± 23.05	251.05 ± 28.82	4.66 (−8.68–18.00)	0.485
Auditory Reaction Time (ms)	SM	235.62 ± 21.86	251.12 ± 16.00	15.50 (6.72–24.28)	0.001
	ST	242.19 ± 21.01	246.81 ± 22.35	4.63 (−4.16–13.41)	0.295

NOTE. Values are presented as mean ± SD. Δ (95% CI) indicates the change from Pre to Post with 95% intervals. *p*-values reflect Pre-to-Post comparisons. SM = social media condition; ST = Stroop task condition; VAS = visual analog scale.

## Data Availability

Data are available upon reasonable request.

## Abbreviations

The following abbreviations are used in this manuscript:

SM	Social media condition
ST	Stroop task condition
VAS	Visual analog scale
RT	Reaction time
VRT	Visual reaction time
ART	Auditory reaction time
LMM	Linear mixed model
